# Automatic and controlled attentional orienting toward emotional faces in patients with Parkinson’s disease

**DOI:** 10.3758/s13415-023-01069-5

**Published:** 2023-02-09

**Authors:** Stefania Righi, Giorgio Gronchi, Silvia Ramat, Gioele Gavazzi, Francesca Cecchi, Maria Pia Viggiano

**Affiliations:** 1 Department of Neuroscience, Psychology, Drug Research and Child’s Health, University of Florence, Via di San Salvi 12, Pad. 26, 50135 Firenze, Italy; 2 Parkinson Unit, Neuromuscular-Skeletal and Sensory Organs Department, AOU Careggi, Firenze, Italy; 3 Department of Experimental and Clinical Medicine, University of Florence, Firenze, Italy; 4 IRCCS Fondazione don Carlo Gnocchi, Firenze, Italy

**Keywords:** Automatic and controlled attention, Emotions, Positivity effect, Dot-probe task, Parkinson’s disease

## Abstract

Parkinson’s disease (PD) is a neurodegenerative motor disorder that can associate with deficits in cognitive and emotional processing. In particular, PD has been reported to be mainly associated with defects in executive control and orienting attentional systems. The deficit in emotional processing mainly emerged in facial expression recognition. It is possible that the defects in emotional processing in PD may be secondary to other cognitive impairments, such as attentional deficits. This study was designed to systematically investigate the different weight of automatic and controlled attentional orienting mechanisms implied in emotional selective attention in PD. To address our purpose, we assessed drug-naïve PD patients and age-matched healthy controls with two dot-probe tasks that differed for stimuli duration. Automatic and controlled attentions were evaluated with stimuli lasting 100 ms and 500 ms, respectively. Furthermore, we introduced an emotion recognition task to investigate the performance in explicit emotion classification. The stimuli used in both the tasks dot-probe and emotion recognition were expressive faces displaying neutral, disgusted, fearful, and happy expressions.

Our results showed that in PD patients, compared with healthy controls, there was 1) an alteration of automatic and controlled attentional orienting toward emotional faces in both the dot-probe tasks (with short and long durations), and 2) no difference in the emotion recognition task. These findings suggest that, from the early stages of the disease, PD can yield specific deficits in implicit emotion processing task (i.e., dot-probe task) despite a normal performance in explicit tasks that demand overt emotion recognition.

**Supplementary Information:**

The online version contains supplementary material available at 10.3758/s13415-023-01069-5.

## Introduction

Parkinson’s disease (PD) is typically characterized as a motor neurodegenerative disorder. PD typically occurs in the aging brain (from 50 years old) and is pathologically characterized by the loss of nigrostriatal dopaminergic innervation (Raket et al., 2022). Alongside the well-known impairments in executive functioning (Dubois & Pillon, 1996), accumulating evidence also suggests deficits in social and emotional processing (Lee et al., 1999; Mitchell & Phillips, 2015; Palmeri et al., 2017; Péron et al., 2012). Those deficits may correlate with global mood state of PD patients increasing the risk of depression and anxiety (Chaudhuri & Naidu, 2008). A recent review (Argaud et al., 2018) suggested that the difficulties of PD patients to infer emotional mental states under ambiguity in everyday situations (Palmeri et al., 2017) may be related to a deficit in facial expression recognition. This deficit seems to involve the whole range of emotions (in particular negative emotions) with greater impact in the advanced stage of PD (Lin et al., 2016; Sprengelmeyer et al., 2003; Suzuki et al., 2006) compared with early stages of the disease (Dujardin et al., 2004; Hipp et al., 2014; Ibarretxe-Bilbao et al., 2009). However, several studies failed to confirm the presence of deficits in emotion processing in PD patients (Adolphs et al., 1998; Albuquerque et al., 2016; Breitenstein et al., 1998; García-Rodríguez et al., 2012; Pell & Leonard, 2005; Wabnegger et al., 2015).

The lack of convergence of the above results could be explained by the dynamic relationship between cognition and emotion. The assumption is that the defects in emotional processing may be secondary to other cognitive impairments, such as attentional deficits. According to the three systems of attention proposed by Posner (alerting, orienting, and executive control systems – Fan et al., 2002, 2005; Posner & Rothbart, 2007), the PD is mainly associated with defects in executive control (Henik et al., 1993; Hsieh et al., 2008) and orienting attentional systems (Cagigas et al., 2007; Cools et al., 2010; Dujardin et al., 2013; Fallon et al., 2016; Horowitz et al., 2006; Machado et al., 2009; Mannan et al., 2008; Posner & Cohen, 1984; Sharpe, 1992). Research that assessed the orienting attention of PD patients by using spatial cueing tasks with nonemotional stimuli (Posner & Cohen, 1984) showed mixed results. Some studies observed normal facilitation effects (Filoteo et al., 1997), whereas other research found increases in distractibility and disruption of the positive compatibility effect (Yamada et al., 1990).

Only a few studies have specifically addressed the relationship between attention and emotion in PD. Garcıa-Rodrıguez and colleagues (2012) investigated divided attention for emotional and neutral faces. The primary task was an emotion recognition task whereas the secondary task was a visuo-spatial short-memory task (Corsi Blocks task). They found in PD patients a worsening of emotional face recognition only when emotional stimuli were processed concurrently with other interfering ones. Exploring a different dimension, Alonso-Recio et al. (2014) found that the visual search process of PD patients was impaired only when they had to search for emotional faces (for all emotional expressions). This study established a relationship between the visual search and deficits in processing of emotional faces, but it did not clarify which component of attention is involved in the impairment shown by the PD patients. Previous research on the time course of orienting attention has shown two partially distinct processes: an early, effortless, automatic stimulus-driven (bottom up) process, that detects the cue (hereafter automatic attention, consistently with Cooper & Langton, 2006) and a slower, effortful, controlled (top-down) process that records the stimulus to be attended (hereafter controlled attention, consistently with Cooper & Langton, 2006; Itti & Koch, 2001; Koch & Ullman, 1985; Weichselgartner & Sperling, 1987). Recently, our previous study (Gronchi et al., 2018), employing two dot-probe tasks, found that automatic and controlled attention differently contribute to the processing of emotional stimuli in healthy elderly people. This study (Gronchi et al., 2018) investigated the “positivity effect,” a well-known, age-related, adaptive mechanism in balancing negative and positive emotions, which transversely involve several cognitive domains, such as working memory (Mikels et al., 2005) and episodic memory (Comblain et al., 2005; Mather & Carstensen, 2003; Scheibe & Carstensen, 2010; Spaniol et al., 2008). The “positivity effect” consists of an attentional preference for positive information as well as avoidance of negative information (Carstensen & Mikels, 2005; Gronchi et al., 2018; Reed & Carstensen, 2012). Gronchi et al. (2018) found that in healthy, elderly people compared with adults the positivity effect is supported by two different attentional mechanisms: the prioritization of positive stimuli depends on automatic attention, whereas the avoidance of negative information depends on controlled attention. Given the different role of automatic and controlled attentional mechanisms in determining the “positivity effect” in healthy elderly people and taking into account that the PD patients showed deficits in both emotion recognition and attention, our aim consists in understanding whether and to what extent the PD may compromise 1) the “positivity effect,” which is commonly observed in the healthy, elderly people, and 2) the automatic and controlled attentional processes involved in orienting toward emotional faces. This topic is doubly relevant. From a theoretical point of view, we propose to clarify the different contributions of automatic and controlled attention to the processing of emotions in PD patients. From a clinical standpoint, we aim to understand whether PD patients balance negative and positive emotions by means of the “positivity effect,” similar to their healthy peers, because this may help to implement interventions to improve the emotional wellbeing and the management of distress in PD patients.

We employed the same procedures of Gronchi et al. (2018). To investigate both the “positivity effect” and automatic and controlled attention toward emotional faces in PD patients, we used two dot-probe tasks with different durations (100 ms and 500 ms). The dot-probe task with short duration (100 ms) allows to investigate the role of automatic attention in the positivity effect (Carstensen & Mikels, 2005; Gronchi et al., 2018; Müller & Rabbitt, 1989; Reed & Carstensen, 2012). By contrast, in the dot-probe task with long duration (500 ms), the positivity effect should be elicited by controlled attentional orienting (Cooper & Langton, 2006). Previous studies that investigated healthy, young subjects with the dot-probe task (Cooper & Langton, 2006) showed the possibility of dissociation in bias toward emotional faces comparing short and long durations. As a matter of fact the pattern of deployment of attention at 100 ms, it can be the opposite of that observed at 500 ms (Cooper & Langton, 2006).

Furthermore, following the study of Linden e collaborators (2010), it is possible to dissociate implicit and explicit emotion processing by means of tasks that require a covert emotion processing (i.e., selective attention tasks that do not require an explicit classification of facial expression, such as the dot-probe task) and an overt classification of the facial expression (i.e., emotion recognition task). Hence, we introduced an emotion recognition task, requesting the subjects to classify the emotional expression to 1) verify the possible presence of emotion recognition deficits in our PD patients and 2) to investigate, in the early stage of PD, the possibility of a dissociation between emotion recognition abilities during implicit tasks that draw on covert emotional processing (i.e., dot-probe task) and explicit tasks that require overt emotion recognition.

## Method

### Recruitment and participants

Given the lowest effect size (η_p_^2^ = 0.050) observed in a previous work similar to this one (Gronchi et al., 2018), a total sample size of 30 participants is sufficient to obtain a power equal to 0.80 with a significance level of 0.05. Inclusion criteria were a specialist’s diagnosis of idiopathic, nondemented, drug-naïve PD and normal or corrected-normal vision. A sample of age-matched healthy controls also was enrolled as a control group.

Thirty-one (15 males) idiopathic, nondemented, drug-naïve PD patients and 33 age-matched, healthy controls (16 males) participated this study. All subjects were right-handed and had a normal or corrected-to-normal vision. Demographical, psychiatric, and disease-related characteristics of the samples are reported in Table 1. There were no differences between controls and PD patients for age, education level, Beck Depression Inventory (BDI-II), State-Trait Anxiety Inventory (STAI), and Mini Mental Status Examination (MMSE) scores (all *p*_*s*_ > 0.05).

**Table 1:** Mean (*M*), standard deviation (SD), range of values, and Student’s *t* test of the demographical, psychiatric, and disease-related characteristics of the samples

	PD patients (*N* = 31)			Controls (*N* = 33)			Student’s *t*
	M	S.D.	Range	M	S.D.	Range	
Age (yr)	65.85	7.56	49-78	66.18	10.05	50-89	t(62) = 0.14, *p* = 0.891
Educational level (yr)	9.21	4.37	5-17	10.32	4.19	4-17	t(62) = −1.02, *p* = 0.312
STAI - trait	37.23	6.69	23-49	36.15	7.43	24-53	t(62) = −0.59, *p* = 0.559
STAI - state	39.23	7.04	26-52	36.94	6.88	21-51	t(62) = −1.29, *p* = 0.201
BDI – II	9.10	6.20	0-21	7.36	4.66	0-19	t(62) = −1.25, *p* = 0.216
MMSE	28.65	1.29	26-30	28.20	1.52	24-30	t(62) = −1.27, *p* = 0.208
Duration of the disease (mo)	7.38	5.43	1-20				
Onset of the disease (age)	65.06	7.47	48-78				
Hoen-Yahr stage	1.59	0.53	1-2				
UPDRS-III	10.70	5.51	3-20				
UPDRS-Total score	15.68	5.95	6-27				

Each participant was evaluated in two distinct, approximately 1-h long, assessment sessions. There was 1 week between the sessions. In the first session, clinical (UPDRS III, Fahn & Elton, 1987; the Hoehn–Yahr Scale, Hoehn & Yahr, 1998), psychiatric (for anxiety: State-Trait Anxiety Inventory – STAI – Spielberger et al., 1983; for depression: Beck Depression Inventory – BDI-II – Beck et al., 1996), and neuropsychological (Mini Mental Status Examination, MMSE – Folstein et al., 1975) data were collected. In the second evaluation session, subjects were administered the experimental procedures. See Supplementary Materials for normality test of the variables and gender comparison. Informed, written consent was obtained from all participants, and ethical approval was obtained.

### Emotion recognition materials

Sixteen face identities (8 females) were taken from the Karolinska Directed Emotional Faces (KDEF) database (Lundqvist et al., 1998). For each identity, the photographs (totaling 64 faces) comprised neutral, disgusted, fearful, and happy expressions. Faces were presented in a grey rectangular frame that measured 8.5 cm by 5.5 cm on the screen. A neutral face was paired with the same identity, displaying one of four emotional expressions: disgusted, fearful, happy, or neutral. The face-pairs were presented on a black background, with one face on the left and the other face on the right, separated by 6 cm.

### Emotion recognition task procedure

The 64 faces (16 face identities with 4 expressions: neutral, disgusted, fearful, and happy) were centrally presented in a random way on a black background for 2,000 ms. Subjects were requested to classify face expression by pressing four buttons on the keyboard. The task was divided in two blocks of 32 faces.

### Dot-probe task procedure

In the dot-probe task, a neutral face was paired with the same identity displaying one of four emotional expressions: angry, fearful, happy, or neutral. The face-pairs were presented on a black background, with one face on the left and the other face on the right, separated by 6 cm.

Two dot-probe tasks with different stimuli durations (SOA) of 100 ms (short duration) and 500 ms (long duration) were run under E-Prime in counterbalanced order across participants. For both dot-probe tasks, the same instructions were given. Participants were told that the task was to identify whether the dots were presented on the left or on the right and that, as such, the faces had nothing to do with the task and should be ignored. Participants had to press one key (v) when the dots were on the left and another key (n) when the dots were on the right.

Each dot-probe task consisted of one block of practice stimuli (3 neutral-neutral picture pairs) followed by eight, randomized, experimental blocks, each containing 28 face-pairs: 24 emotional-neutral face-pairs (8 disgusted-neutral, 8 fearful-neutral, and 8 happy-neutral, of which 12 were congruent and 12 were incongruent), and four neutral-neutral face pairs for a total of 224 face-pair presentations. Congruent and incongruent mean that the emotional face and the dot appear in the same or in the opposite location, respectively. Each emotional-neutral face-pair was randomly presented four times with an equal number of both congruent and incongruent probe presentations and left vs. right locations. The other 32 neutral-neutral pairs of faces of the same identity were included to act as a baseline to control for which mechanisms (i.e., facilitation or inhibition) might be responsible for any observed attentional biases (Koster et al., 2004). Specifically, responses faster than the baseline (neutral-neutral pairs of faces) would indicate that facilitation (vigilance) was taking place at that location compared with baseline responding. Responses slower than the baseline would indicate inhibition (avoidance) at that location compared with baseline responding (Koster et al., 2004, 2005).

**Fig. 1:**
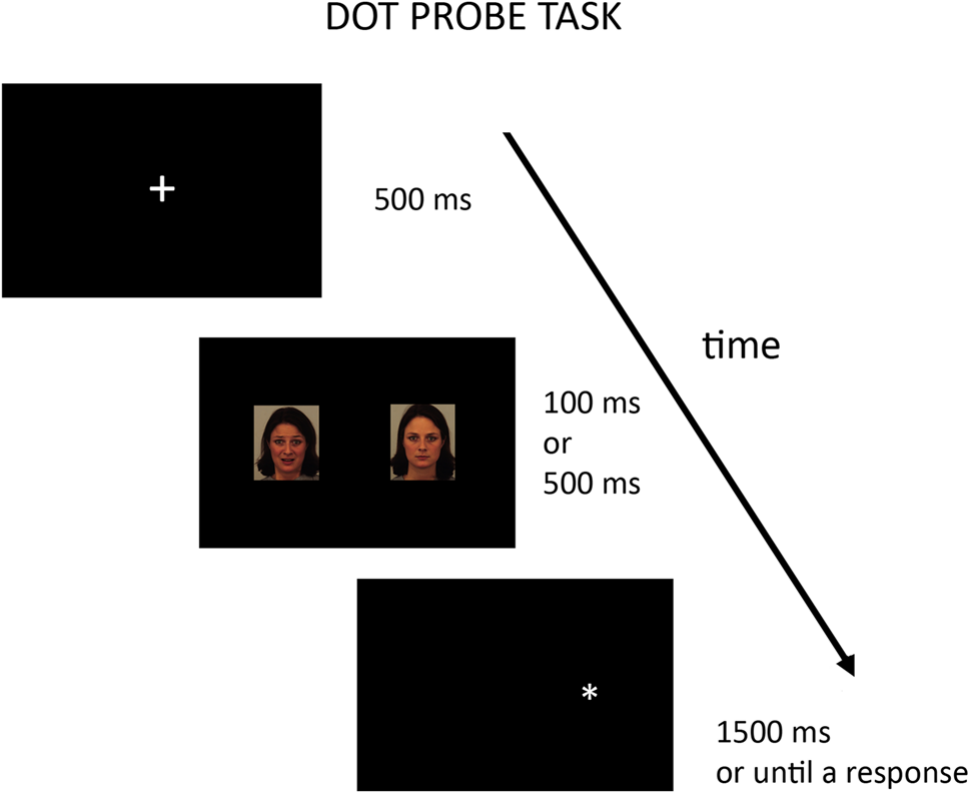
Dot-probe task

Each dot-probe task was composed of 28 trials that consisted of three sequential components: 1) a central white fixation cross (500 ms); 2) a 100- or 500-ms simultaneous presentation of two faces (face-pairs) located immediately to the left and to the right of the fixation cross; and 3) a white asterisk (i.e., dot-probe) appearing in either the left or right location immediately after the offset of the faces (Fig. 1).

### Data analysis

With regard to the emotion recognition task, the proportion of corrected recognition (accuracy) and mean reaction times (RTs) for correct responses were separately analyzed by using two repeated measures ANOVA with 2 levels Group (controls vs. PD patients) and 4 levels of Emotional face (neutral, disgusted, fearful, and happy).

With regard to the dot-probe task, reaction times shorter than 200 ms were removed, given that it is the minimum estimated time to identify a visually presented object (Johnson, 2010, see also Gronchi & Sloman, 2021; Mather & Carstensen, 2003). Long reaction times were excluded for the heavy-tailed distribution of reaction times (Mather & Carstensen, 2003; Ratcliff, 1993). Furthermore, individual outliers (defined as RTs that deviated more than three SDs from the individual mean latency time) also were discarded. Because a preliminary analysis (ANOVA) revealed no main effect or interaction of picture position (left vs. right), RTs were collapsed across the factor picture position. Following previous research (Cooper & Langton, 2006; Gronchi et al., 2018; Koster et al., 2004, 2005), we conducted repeated measure ANOVAs on the accuracy and reaction times data, followed by post-hoc comparisons with the Bonferroni correction. Thus, the proportion of corrected responses (accuracy) and mean RTs were analyzed by means of a repeated ANOVA with 2 levels of Duration (100 ms vs. 500 ms), 3 levels of Emotional face (disgusted, fearful and happy), 2 levels of Congruency (congruent vs. incongruent), and 2 levels of Group (controls vs. PD patients).

Following previous works (Gronchi et al., 2018; Koster et al., 2005; Linden et al., 2010), individual Attentional Bias Indexes (ABIs) and Attentional Facilitation Indexes (AFIs) were computed. ABI was calculated by subtracting mean RTs on congruent trials from mean RTs on incongruent trials for each type of emotional face-pair: positive ABI values reflect attention toward the emotional face (vigilance), and negative values reflect attention away from the emotional face (avoidance). The AFI was computed by subtracting from the baseline RTs of the trials of neutral-neutral face-pairs the mean of each of the three congruent emotional-neutral conditions. Positive AFI values indicate that facilitation (attentional capture) was due to the congruent emotional location, whereas negative AFI values would suggest inhibition (avoidance) of congruent emotional locations compared with neutral baseline responses (Koster et al., 2005). ABIs and AFIs were analyzed by means of a repeated measure ANOVA with 2 levels of Duration (100 ms vs. 500 ms), 3 levels of Emotional face (disgusted, fearful and happy), and 2 levels of Group (controls vs. PD patients).

For each analysis, a normality test (Kolmogorov-Smirnov) was conducted on the dependent variables (see Supplementary Materials). As expected, being accuracy in both tasks (emotion recognition and dot-probe) nearly at-ceiling, normality was violated. In a similar manner, being reaction times rightly skewed, normality was violated (although with a negligible effect for dot-probe RTs). Given the robustness of ANOVA to normality violation (Blanca Mena et al., 2017), previously described analyses were performed. ABI and AFI were normally distributed.

## Results^1^

### Emotion recognition task

**Fig. 2:**
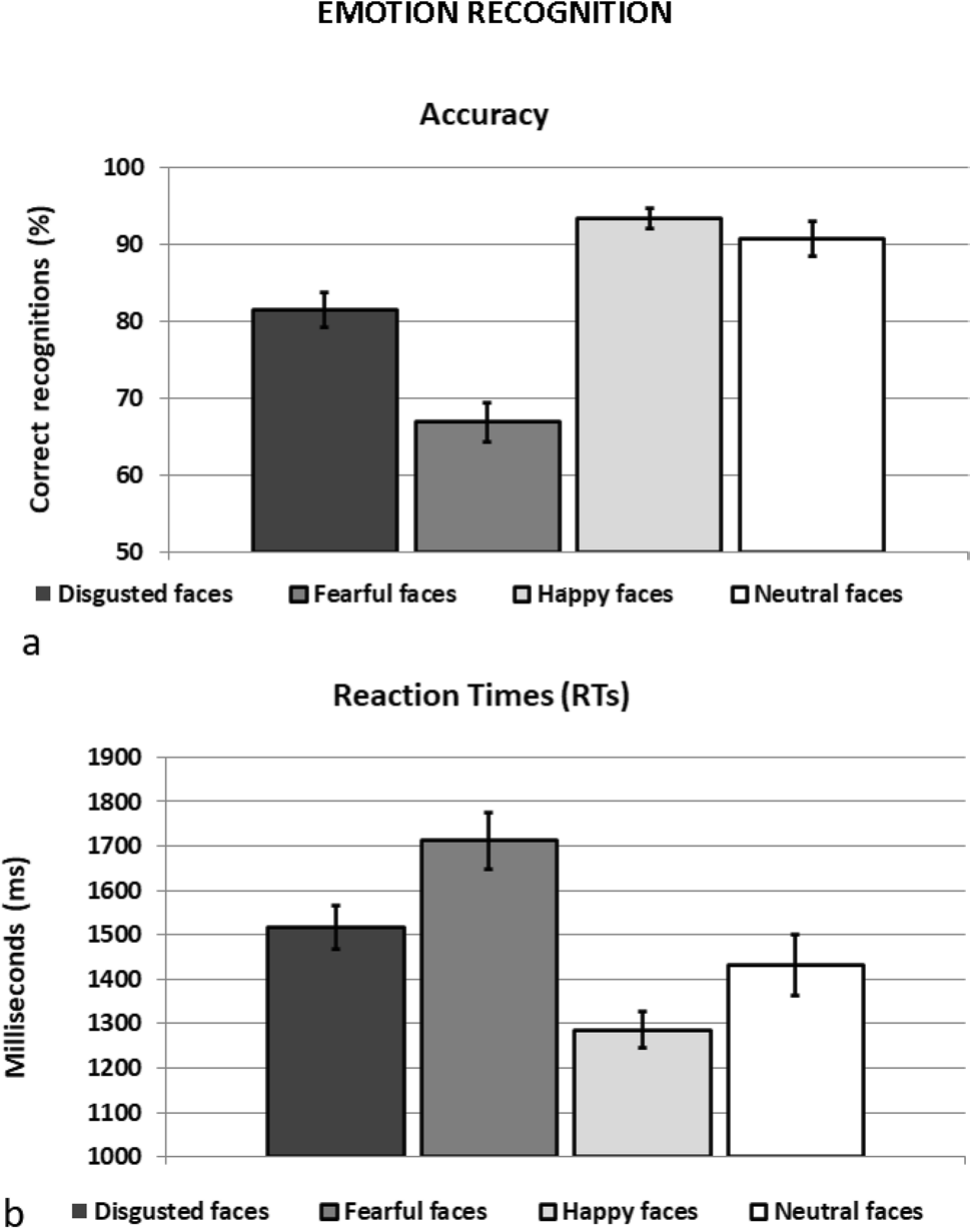
Recognition task performance for both groups controls and PD patients together (because no main or interaction effect of Group was found). **a** Accuracy: proportion of correct responses; **b** Mean reaction times (RTs) in milliseconds. Error bars represent standard error (SE)

The ANOVA on accuracy revealed a significant main effect of Emotional face, F(3, 186) = 39.87, *p* < 0.001, η_p_^2^ = 0.391. Post-hoc comparisons with Bonferroni’s correction showed that fearful expression was less recognized than neutral (*p* < 0.001), disgusted (*p* < 0.001), and happy expression (*p* < 0.001). Furthermore, disgusted expression was less recognized than neutral (*p* = 0.004) and happy expression (*p* < 0.001; Fig. 2a). No significant differences emerged for the Group variable, F(1, 62) = 0.03, *p* = 0.868, η_p_^2^ = 0.001 and for the interaction Emotion x Group, F(3, 186) = 0.03, *p* = 0.823, η_p_^2^ = 0.005.

Consistently, the ANOVA on the RTs showed a significant main effect of Emotional face, F(3, 186) = 18.60, *p* < 0.001, η_p_^2^ = 0.23. The RTs for fearful expression were slower with respect to neutral (*p* = 0.003), disgusted (*p* = 0.001), and happy expression (*p* < 0.001). The RTs for happy expression were faster with respect to neutral (*p* = 0.018) and disgusted faces (*p* < 0.001; Fig. 2b). No significant differences for the Group variable, F(1, 62) = 0.01, *p* = 0.916, η_p_^2^ = 0.001 and for the interaction Emotion x Group, F(3, 186) = 0.16, *p* = 0.925, η_p_^2^ = 0.003.

### Dot-probe task

With regard to the accuracy of the dot-probe task, the only statistically significant effect was the Duration F(1, 62), = 4.44, *p* = 0 .039, η_p_^2^ = 0.003, where greater accuracy was observed in the 500-ms condition compared with the 100-ms condition. The remaining main effects—Emotion, F(1, 62) = 1.44, *p* = 0.235, η_p_^2^ = 0.023; Group, F(1, 62) = 0.190, *p* = 0.666, η_p_^2^ = 0.003; and Congruency, F(1, 62) = 0.300, *p* = 0.585, η_p_^2^ = 0.005—were not statistically significant.

**Fig. 3:**
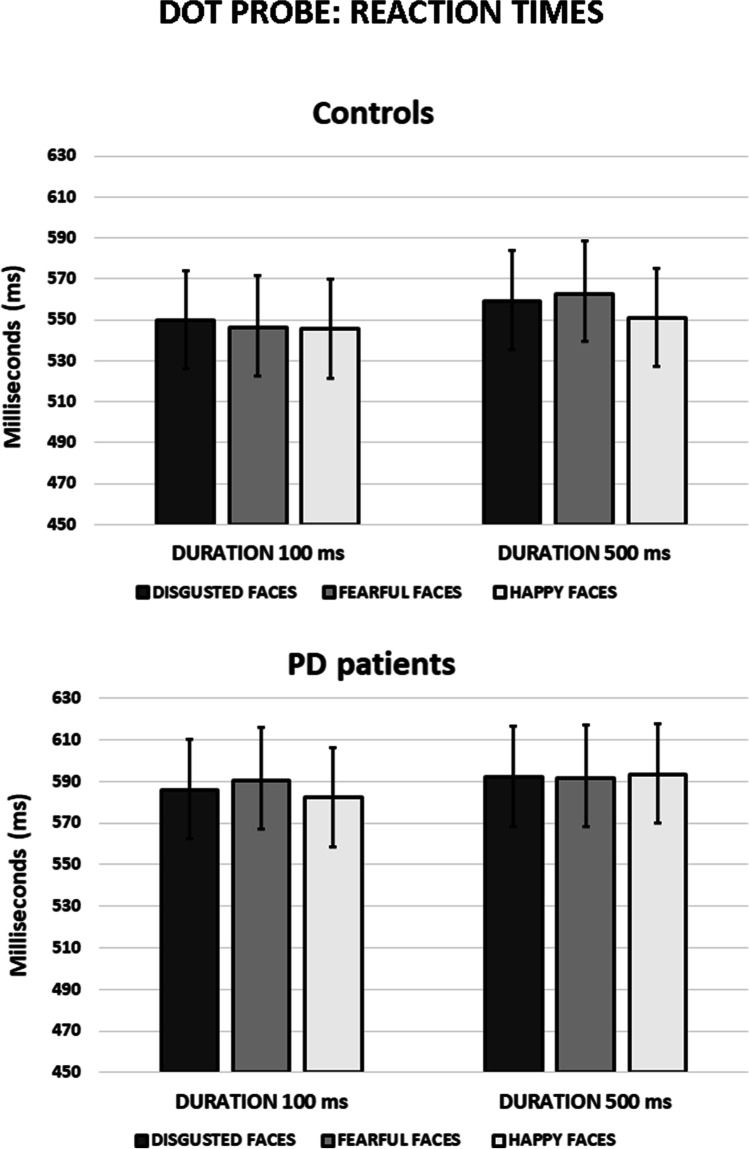
Dot probe task performance in controls and PD patients. Mean reaction times for dot-probe tasks with short (100 ms) and long duration (500 ms). Error bars represent standard error (SE)

With regard to RTs, the significant main effect of Emotional face, F(2, 124) = 3.92, *p* = 0.022, η_p_^2^ = 0.059, evidenced longer RTs for fearful expression compared with happy (p = 0.049) (means = 572.58 and 568.03, respectively). The significant main effect of Congruency, F(1, 62) = 4.36, *p* = 0.041, η_p_^2^ = 0.066, revealed longer RTs for incongruent compared with congruent trials (means = 572.28 and 569.22, respectively). The main effect of Group was not significant, F(1, 62) = 1.77, *p* = 0.188, η_p_^2^ = 0.028. Furthermore, the interactions Duration x Emotional face x Group, F(2, 124) = 4.22, *p* = 0.017, η_p_^2^ = 0.064 and Duration x Emotional face x Congruency, F(2, 124) = 5.27, *p* = 0.006, η_p_^2^ = 0.078, were statistically significant Fig. 3.

The following results about the ABI analysis allow better understanding of these three-way significant interactions. In particular, analyzing ABI values, we observed the significant main effect of Emotional face, F(2, 124) = 3.74, *p* = 0.027, η_p_^2^ = 0.057, indicating that more attention was devoted to happy faces with respect to fearful faces. The main effect of Duration, F(1, 62) = 2.41, *p* = 0.126, η_p_^2^ = 0.037 and the main effect of Group F(1, 62) = 1.79, *p* = 0.186, η_p_^2^ = 0.028 were not significant. The significant interaction Duration x Emotional face, F(2, 124) = 3.78, *p* = 0.025, η_p_^2^ = 0.057, was further qualified by the significant interaction Duration x Emotional face x Group, F(2, 124) = 3.89, *p* = 0.023, η_p_^2^ = 0.059.

**Fig. 4:**
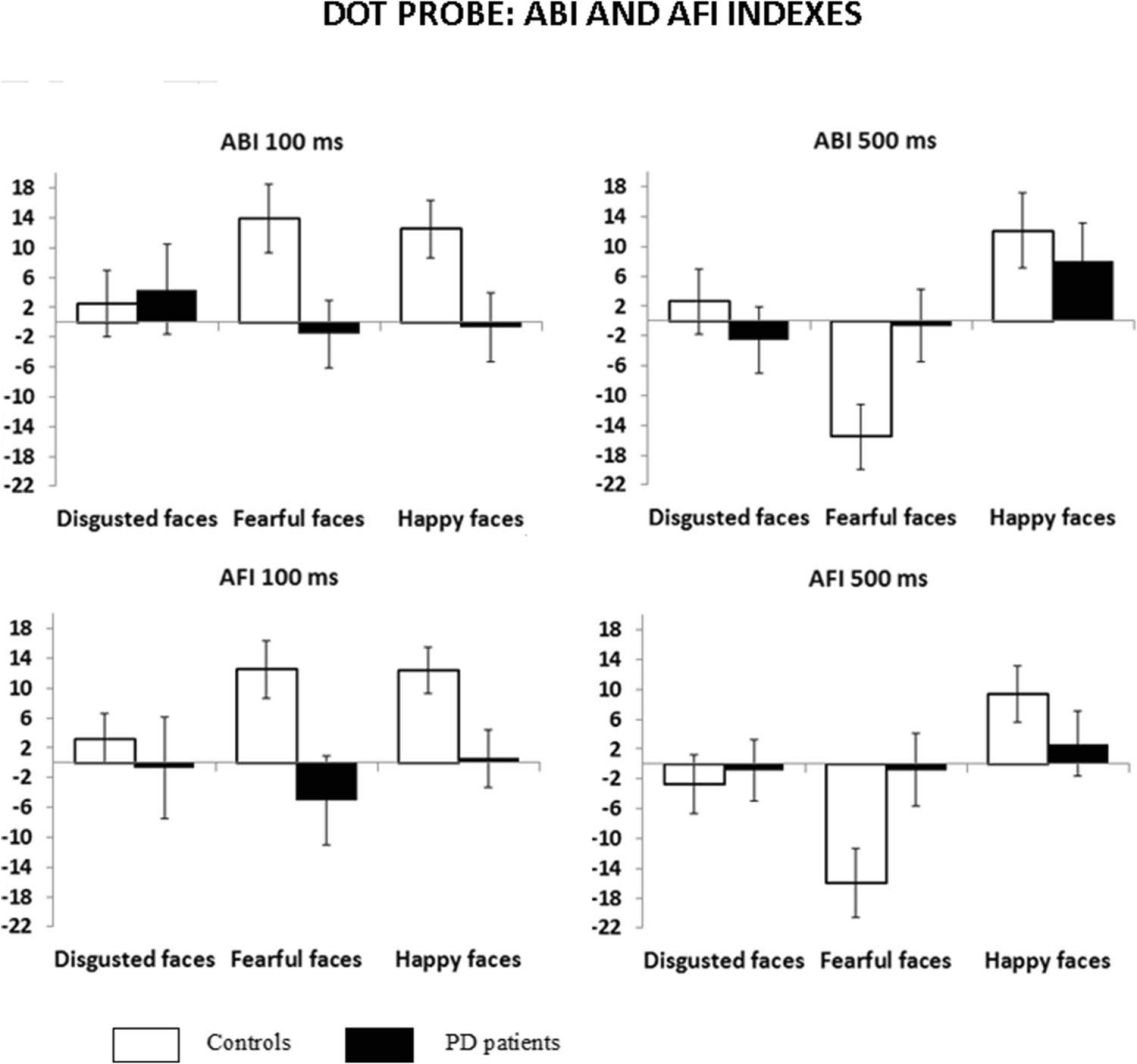
Dot-probe task performance in controls and PD patients. Attentional bias index (ABI) and Attentional Facilitation Index (AFI) for dot-probe tasks with short (100 ms) and long duration (500 ms). Positive ABI values reflect attention toward the emotional face (vigilance), and negative values reflect attention away from the emotional face (avoidance). Positive AFI values indicate that facilitation (attentional capture) was due to the congruent emotional location, whereas negative AFI values would suggest inhibition (avoidance) of congruent emotional locations compared with neutral baseline responses. Error bars represent standard error (SE)

Post-hoc comparison (computed with Bonferroni’s correction) revealed that at a duration of 100 ms, the PD patients allocated less attention to both the fearful (*p* = 0.021) and the happy faces (*p* = 0.035) than the controls. At 500-ms duration, the PD patients compared with controls did not divert attention from fearful faces (*p* = 0.029; Fig. 4).

The AFI results evidenced the significant main effect of Emotional face, F(2, 124) = 5.69, *p* = 0.004, η_p_^2^ = 0.084, revealing that happy faces captured more attention with respect to disgusted (*p* = 0.014) and fearful faces (*p* = 0.014). The main effect of Duration, F(1, 62) = 2.14, *p* = 0.149, η_p_^2^ = 0.033 and the main effect of Group, F(1, 62) = 1.45, *p* = 0.233, η_p_^2^ = 0.023 were not statistically significant. Furthermore, the significant interactions Duration x Group, F(1, 62) = 4.13, *p* = 0.047, η_p_^2^ = 0.062, was further qualified by the significant interaction Duration x Emotional face x Group, F(2, 118) = 4.64, *p* = 0.011, η_p_^2^ = 0.070. Post-hoc comparison, showed that in the 100-ms condition, the PD patients were not facilitated by both the fearful (*p* = 0.016) and the happy faces (*p* = 0.023) compared with controls. At a duration of 500 ms, the PD patients did not divert their attention away from the fearful faces as instead do the controls (*p* = 0.031). Furthermore, at 500-ms duration within the controls, the AFI for fearful faces was lower with respect to both the happy (*p* = 0.001) and the disgusted faces (*p* = 0.021), whereas within the PD patients did not emerge differences (Fig. 4).

The automatic attentional orienting of controls was captured by negative threat (fearful) and positive (happy) expressions, whereas the PD patients did not pay preferential automatic attention to emotional faces. As pertains voluntary attention, at 500-ms duration, the PD patients did not avoid fearful expression differently from the controls. PD patients at 500 ms did not differ from controls in processing happy faces.

## Discussion

The present study systematically investigated the different weight of automatic and controlled attentional processes involved in orienting toward emotional faces in PD patients. Furthermore, our aim was to verify whether, and to what extent, PD patients balance negative and positive emotions through the “positivity effect” that is an attentional bias typically observed in healthy elderly people (Carstensen & Mikels, 2005; Gronchi et al., 2018; Reed & Carstensen, 2012). Our results highlighted that in PD patients 1) the typical age-related “positivity effect” was lacking, and 2) both automatic and controlled attentional orienting toward emotional faces were altered.

Specifically, we found that automatic attentional orienting of PD patients (at 100 ms) was not captured by emotional faces (fearful and happy) as it was in healthy controls. In addition, an abnormal attentional bias in the controlled attentional orienting (at 500 ms) also was observed in PD patients: at 500-ms duration the PD patients, differently from controls, did not avoid the fearful expressions. Because the two groups did not differ in psychiatric tests, these differences cannot be attributed to different levels of affective dysfunction, at least for what pertains to anxiety or depression. Furthermore, the lack of difference between groups in the recognition of emotional faces makes it difficult to attribute our results to deficits in emotion recognition. In fact, both groups (controls and PD patients) showed a worse performance (in RTs and accuracy) for fearful faces with respect to all the other expressions, and a better performance for happy faces with respect to all the other expressions. Taking this into account, our findings would suggest that from the early stages of the disease, PD can yield specific deficits in implicit emotion processing task (i.e., dot-probe task) despite a normal performance in explicit tasks that demand overt emotion recognition (i.e., classification of emotional expressions). Our previous study (Gronchi et al., 2018), which employed the dot-probe task, showed that two different attentional processes are responsible for the age-related positivity effect: an automatic attentional bias toward positive stimuli, and a controlled attentional mechanism that diverts attention away from negative stimuli.^2^ Neuroimaging studies (Iidaka et al., 2002; Mather et al., 2004) showed in healthy elderly people a dissociation with reduced activity in the left amygdala and the right parahippocampal gyrus. This may suggest that aging differentially affects neural responses to faces with negative or positive emotional expressions (Calder et al., 2003). Specifically, these age-related, neural changes might contribute to the typical “positivity effect” that prioritize the automatic processing of positive over negative emotional information (Calder et al., 2003; Williams et al., 2006).

The lack of the “positivity effect” that we observed in PD subjects might be associated with the impaired dopamine transmission in the mesocorticolimbic pathway, which compromises orbitofrontal and amygdalar presynaptic dopaminergic functions in the early stages of PD (Lotze et al., 2009; Ouchi et al., 1999). In fact, PD is pathologically characterized by the loss of nigrostriatal (Dagher et al., 2001; Owen et al., 1998) and mesocortical (Cools et al., 2002; Mattay et al., 2002) dopaminergic circuits. Specifically, degeneration of dopamine producing neurons in the substantia nigra pars compacta and the putamen-caudate complex leads to diminished concentrations of dopamine in the nigrostriatal pathway and prefrontal cortex (Gröger et al., 2014; Narayanan et al., 2013). Discharge of the dopaminergic neurons at mesencephalic level correlated with attentional processes in behaving animals (Amalric et al., 1995; Apicella et al., 1991; Montaron et al., 1982), in ADHD children (Wu et al., 2012) and in normal controls (fMRI evidence on attentional orienting) (Anderson, 2013; Anderson et al., 2016). Particularly, the orienting of attention toward salient stimuli is positively correlated with the release of dopamine within the caudate and posterior putamen (Anderson et al., 2011; Anderson et al., 2016).

Furthermore, although the amygdala is not part of the frontostriatal circuitry, several studies found in PD off dopaminergic medications, a hypoactivation of amygdala in both cognitive (Kim et al., 2018) and emotional saliency appraisal tasks (Argaud et al., 2018; Diederich et al., 2016; Tessitore et al., 2002; Yoshimura et al., 2005). Basically, amygdala activity is likely a neural mechanism involved in automatic vigilance/facilitated attention for emotional information, especially when it is threat-related (Carlson et al., 2009; Davis & Whalen, 2001; Öhman, 2005). On the basis of this reasoning, we hypothesised that defects in the dopaminergic functioning by affecting amygdala and mesocorticolimbic pathway might compromise the automatic attentional orienting toward fearful faces and happy faces in PD. This lack of attentional orienting in an implicit emotional task (that implies a covert emotion processing), such as the dot probe task, is consistent with previous neuroimaging evidence of reduced amygdala responses in patients with PD, in drug-off state, during the perceptual processing of angry and fearful faces (Tessitore et al., 2002). The role of dopamine depletion in the deficit exhibited by patients with PD also is supported by a recent neuroimaging study by Frick et al. (2022), which showed that striatal dopamine release in the amygdala is fundamental in fear memory formation; the dopamine release reflects attentional orienting, fundamental for anticipation of salient stimuli. Hence, for fearful faces, PD damages the evolutionary adaptive capacity to orient automatic attention to anticipate salient stimuli, which may represent o signal a danger (threat-related stimuli). Similarly, PD prejudices the automatic attentional orienting toward happy faces, which represents a salient stimulus relevant for social interaction and social reward (Park et al., 2018).

During the controlled phase of emotional stimuli evaluation, PD patients did not divert their attention from fearful faces, whereas elderly controls selectively avoided negative threat-related information to reduce the distress. From the evolution perspective and adaptation to environment, repeated emotional experiences over the lifespan lead humans to be more selective for input providing positive outcomes (Carstensen & Mikels, 2005). Our data suggested that PD can alter this adaptive mechanism through a selective impairment in the ability to divert controlled attention from negative threat-related information. On the contrary, the controlled allocation of attention toward happy stimuli, although not identical to controls, appears to be less altered. This impairment in controlled attentional allocation is consistent with previous research that showed that the depletion of striatal dopamine in the basal ganglia can compromise conscious attention (Slagter et al., 2016) and cognitive control during action selection (Wylie et al., 2010). The deficit for controlled attentional orienting toward fearful faces may be related to the evidence that the pattern of dopaminergic depletion in PD may be greater for the specific regions of the amygdala, insula, and the orbitofrontal and the anterior cingulate cortices that subserve the recognition of negative emotions, especially when threat-related (Diano et al., 2017; Murphy et al., 2003; Ruffman et al., 2008; Vytal & Hamann, 2010). Nonetheless, although disgust can be considered a threat-related emotion (Davey, 2011), we did not find any attentional bias for disgusted faces in both PD patients and controls, consistently with previous work (Gronchi et al., 2018). These results may be related to the neurophysiological evidence that as early as 96 ms after stimulus presentation, sensory perceptual, and attentional processing of disgust differ from fear information (Krusemark & Li, 2011). Consistent with our results, Krusemark and Li (2011) showed that increased attention and facilitated visual search performance are boosted by fearful stimuli, consistently with the well-documented role of fear in enhancing information processing, because fear is a direct response to threat (Dolan, 2002; LeDoux, 2000; Phelps, 2006). On the other hand, disgust stimuli suppressed visual attention to the degree that they impeded search of the target; this is in accord with the evolutionary function of disgust of minimizing exposure to a possible contamination and/or poisoning (Davey, 2011; Krusemark & Li, 2011; Rozin & Fallon, 1987).

As it pertains to the explicit emotion recognition task, consistent with previous research, no differences between PD patients and controls emerged (Adolphs et al., 1998; Breitenstein et al., 1998; Pell & Leonard, 2005; Suzuki et al., 2006). Thus, it could be the case that at early stages of PD disease, patients may have no impairment in simple emotion recognition if all cognitive resources are devoted to a single task. However, the alteration that we found in the covert attentional orienting toward some emotions may compromise the emotional regulation and social cognition in everyday life (Palmeri et al., 2017).

The main limitation of the present study is to have excluded PD patients in the drug-on state. This would allow to verify the motor speed in the RTs with respect to drug-naïve patients and to attribute the deficit that we observed entirely to the dopamine depletion in basal ganglia circuits more reliably. Another limitation concerns the restricted exploration of attention to its controlled and automatic submodalities. Future studies investigating aspects of attention that we did not take into consideration will be necessary both to confirm our results and to deeply evaluate attentional deficits in PD patients during the emotional faces processing.

In light of the results obtained, this study could be a starting point to deepen the investigation on the emotional functioning in PD subjects and to implement rehabilitative interventions to improve emotional and social life of PD patients.

## Supplementary information

ESM 1 (DOCX 89 kb)

## Footnotes

1 The complete output tables, along with descriptive statistics, are reported in the Supplementary Materials file.

2 The automatic prioritization of positive stimuli on automatic attention and the controlled avoidance of negative information apparently seem to not reflect the results reported in Figure 3 where the healthy controls 1) have positive ABI and AFI indexes at 100 ms for both happy and fearful; 2) at 500 ms ABI/AFI are negative and positive for fearful and happy faces, respectively. It should be noted that the positivity effect emerges with respect to the comparison between healthy elderly and healthy adults. Indeed, the results in the control group of healthy elderly people of this paper replicate the results observed in an equivalent healthy elderly sample of our previous study (Gronchi et al., 2018; Figure 2).
