# Supplementary material for: Automatic and controlled attentional orienting toward emotional faces in patients with Parkinson’s disease
Source: Cogn Affect Behav Neurosci. 2023 Feb 9;23(2):371–82. doi: 10.3758/s13415-023-01069-5 (PMC10050058; doi:10.3758/s13415-023-01069-5)
Supplement: Supplementary file 1 — (DOCX 89 kb) [file 13415_2023_1069_MOESM1_ESM.docx]

**Supplementary materials**

**Automatic and controlled attentional orienting toward emotional faces in PD patients**

**Sample:**

- Control group: n = 33
- PD patients: n = 31

**Normality check (variables reported in Table 1)**

| **Variable** | **Group** | **Kolmogorov-Smirnov Test (D)** | **p-value** |
| --- | --- | --- | --- |
| Education | Control | .22 | .08 |
|  | PD | .22 | .06 |
| Age | Control | .16 | .37 |
|  | PD | .13 | .61 |
| BDI-2 | Control | .12 | .68 |
|  | PD | .10 | .89 |
| STAI-S | Control | .11 | .81 |
|  | PD | .13 | .64 |
| STAI-T | Control | .11 | .81 |
|  | PD | .11 | .84 |
| MMSE | Control | .19 | .18 |
|  | PD | .21 | .11 |
| Duration disease (months) | PD | .21 | .13 |
| Onset disease (age) | PD | .17 | .27 |
| Hoehn and Yahr | PD | .30 | .001 |
| UPDRS III | PD | .16 | .38 |
| UPDRS TOTAL | PD | .11 | .84 |

**Male-Female comparisons (variables reported in Table 1)**

Educational level

| **Group** | **Gender** | **Mean** | **Standard Deviation** |
| --- | --- | --- | --- |
| Control | Male | 11.06 | 4.63 |
|  | Female | 7.47 | 3.56 |
| PD | Male | 10.27 | 4.15 |
|  | Female | 10.38 | 4.50 |

| **Effect** | **F(df1,df2)** | **p-value** | **η_p_^2^** |
| --- | --- | --- | --- |
| Group | F(1,60) = 1.00 | .322 | .016 |
| Gender | F(1,60) = 2.72 | .104 | .043 |
| Group*Gender | F(1,60) = 3.07 | .085 | .049 |

Age

| **Group** | **Gender** | **Mean** | **Standard Deviation** |
| --- | --- | --- | --- |
| Control | Male | 67.38 | 9.08 |
|  | Female | 65.06 | 11.33 |
| PD | Male | 66.80 | 7.51 |
|  | Female | 65.00 | 7.95 |

| **Effect** | **F(df1,df2)** | **p-value** | **η_p_^2^** |
| --- | --- | --- | --- |
| Group | F(1,60) = .02 | .890 | <.001 |
| Gender | F(1,60) = .81 | .373 | .013 |
| Group*Gender | F(1,60) = .01 | .911 | <.001 |

BDI-II

| **Group** | **Gender** | **Mean** | **Standard Deviation** |
| --- | --- | --- | --- |
| Control | Male | 5.69 | 3.63 |
|  | Female | 8.94 | 5.19 |
| PD | Male | 9.13 | 6.15 |
|  | Female | 9.06 | 6.65 |

| **Effect** | **F(df1,df2)** | **p-value** | **η_p_^2^** |
| --- | --- | --- | --- |
| Group | F(1,60) = 1.67 | .201 | .027 |
| Gender | F(1,60) = 1.33 | .253 | .022 |
| Group*Gender | F(1,60) = 1.45 | .233 | .024 |

STAI-S

| **Group** | **Gender** | **Mean** | **Standard Deviation** |
| --- | --- | --- | --- |
| Control | Male | 33.56 | 6.38 |
|  | Female | 40.12 | 6.12 |
| PD | Male | 39.73 | 6.54 |
|  | Female | 38.75 | 7.87 |

| **Effect** | **F(df1,df2)** | **p-value** | **η_p_^2^** |
| --- | --- | --- | --- |
| Group | F(1,60) = 2.02 | .161 | .033 |
| Gender | F(1,60) = 2.72 | .105 | .043 |
| Group*Gender | F(1,60) = 4.97 | .030 | .077 |

STAI-T

| **Group** | **Gender** | **Mean** | **Standard Deviation** |
| --- | --- | --- | --- |
| Control | Male | 35.56 | 7.68 |
|  | Female | 36.71 | 7.61 |
| PD | Male | 37.73 | 6.80 |
|  | Female | 36.75 | 7.51 |

| **Effect** | **F(df1,df2)** | **p-value** | **η_p_^2^** |
| --- | --- | --- | --- |
| Group | F(1,60) = .36 | .553 | .006 |
| Gender | F(1,60) = .003 | .966 | <.001 |
| Group*Gender | F(1,60) = .33 | .569 | .005 |

MMSE

| **Group** | **Gender** | **Mean** | **Standard Deviation** |
| --- | --- | --- | --- |
| Control | Male | 28.50 | 1.21 |
|  | Female | 27.91 | 1.79 |
| PD | Male | 28.77 | 1.35 |
|  | Female | 28.54 | 1.26 |

| **Effect** | **F(df1,df2)** | **p-value** | **η_p_^2^** |
| --- | --- | --- | --- |
| Group | F(1,60) = 1.57 | .215 | .026 |
| Gender | F(1,60) = 1.30 | .259 | .021 |
| Group*Gender | F(1,60) = .26 | .614 | .004 |

PD patients

| **Variable** | **Gender** | **Mean** | **Standard Deviation** |
| --- | --- | --- | --- |
| Duration of disease (months) | Male | 6.67 | 5.74 |
|  | Female | 8.06 | 6.40 |
| Onset disease (age) | Male | 66.07 | 7.41 |
|  | Female | 64.13 | 7.87 |
| Hoen-Yahr stage | Male | 1.57 | .46 |
|  | Female | 1.59 | .49 |
| UPDRS-III | Male | 10.13 | 4.63 |
|  | Female | 11.25 | 6.51 |
| UPDRS-Total score | Male | 15.07 | 5.13 |
|  | Female | 16.25 | 6.92 |

| **Variable** | **t(df)** | **p-value** |
| --- | --- | --- |
| Duration of disease (months) | t(29) = -.638 | .529 |
| Onset disease (age) | t(29) = .706 | .486 |
| Hoen-Yahr stage | t(29) = -.159 | .875 |
| UPDRS-III | t(29) =-.547 | .588 |
| UPDRS-Total score | t(29) = -0.538 | .595 |

**EMOTION RECOGNITION TASK**

**Accuracy**

Normality check: D = .20, p < .001

| **Emotion** | **Group** | **Mean accuracy** | **Standard Deviation** | **95% IC** |
| --- | --- | --- | --- | --- |
| Disgusted | Control | .81 | .12 | .75 .87 |
|  | PD | .82 | .23 | .76 .89 |
| Fear | Control | .66 | .18 | .59 .73 |
|  | PD | .68 | .23 | .60 .75 |
| Happy | Control | .95 | .07 | .91 .99 |
|  | PD | .92 | .14 | .88 .96 |
| Neutral | Control | .90 | .20 | .84 .96 |
|  | PD | .92 | .16 | .85 .98 |

| **Effect** | **F(df1,df2 )** | **p-value** | **η_p_^2^** |
| --- | --- | --- | --- |
| Emotion | F(3, 186) = 39.87 | < .001 | .391 |
| Group | F(1, 62) = .03 | .868 | .001 |
| Emotion*Group | F(3, 186) = .30 | .823 | .005 |

**Main Effects (accuracy)**

| **Group** | **Mean accuracy** | **Standard Error** | **Mean Difference (Standard Error) and p-value** |
| --- | --- | --- | --- |
| Control Group | .83 | .019 | -.005 (.028), p = .868 |
| PD | .83 | .020 |  |

| **Emotion** | **Mean accuracy** | **Standard**  **Error** | **Comparison: Mean Difference (Standard Error) and p-value** |
| --- | --- | --- | --- |
| Disgusted | .82 | .023 | vs Fear: .146 (.029), p < .001  vs Happy: -.118 (.025), p < .001  vs Neutral: -.092 (.026), p = .004 |
| Fear | .67 | .025 | vs Happy: -.265 (.025), p < .001  vs Neutral: -.239 (.033), p < .001 |
| Happy | .93 | .014 | vs Neutral: .026 (.022), p = 1.00 |
| Neutral | .91 | .022 |  |

**Reaction Times**

Normality check: D = .14, p < .001

| **Emotion** | **Group** | **Mean RTs** | **Standard Deviation** | **95% IC** |
| --- | --- | --- | --- | --- |
| Disgusted | Control | 1535 | 441 | 1395 1676 |
|  | PD | 1499 | 359 | 1354 1643 |
| Fear | Control | 1696 | 585 | 1514 1878 |
|  | PD | 1730 | 445 | 1543 1918 |
| Happy | Control | 1301 | 366 | 1184 1418 |
|  | PD | 1269 | 300 | 1148 1389 |
| Neutral | Control | 1434 | 684 | 1243 1626 |
|  | PD | 1431 | 358 | 1233 1628 |

| **Effect** | **F(df1,df2 )** | **p-value** | **η_p_^2^** |
| --- | --- | --- | --- |
| Emotion | F(3, 186) = 18.60 | < .001 | .231 |
| Group | F(1, 62) = .01 | .916 | .001 |
| Emotion*Group | F(3, 186) = .16 | .925 | .003 |

**Main Effects (reaction times)**

| **Group** | **Mean RTs** | **Standard Error** | **Mean Difference (Standard Error) and p-value** |
| --- | --- | --- | --- |
| Control Group | 1492 | 63 | 9.59 (90), p = .916 |
| PD | 1482 | 65 |  |

| **Emotion** | **Mean RTs** | **Standard**  **Error** | **Comparison: Mean Difference (Standard Error) and p-value** |
| --- | --- | --- | --- |
| Disgusted | 1517 | 51 | vs Fear: -196 (50), p = .001  vs Happy: 232 (46), p < .001  vs Neutral: 84 (73), p = 1.00 |
| Fear | 1713 | 65 | vs Happy: 428 (52), p < .001  vs Neutral: 281 (75), p = .003 |
| Happy | 1285 | 42 | vs Neutral: -.148 (48), p = .018 |
| Neutral | 1433 | 69 |  |

**DOT-PROBE TASK**

**Accuracy**

Normality check: D = .38, p < .001

| **Emotion** | **Group** | **Duration (ms)** | **Congruence** | **Mean accuracy** | **Standard Deviation** | **95% CI** |
| --- | --- | --- | --- | --- | --- | --- |
| Disgusted | Control | 100 | Congruent | .97 | .06 | .94 1.00 |
|  |  |  | Incongruent | .97 | .05 | .95 1.00 |
|  |  | 500 | Congruent | .99 | .02 | .99 1.00 |
|  |  |  | Incongruent | .99 | .03 | .98 1.00 |
|  | PD | 100 | Congruent | .97 | .11 | .94 1.00 |
|  |  |  | Incongruent | .97 | .10 | .94 .99 |
|  |  | 500 | Congruent | 1.00 | .01 | .99 1.00 |
|  |  |  | Incongruent | .99 | .02 | .99 1.00 |
| Fear | Control | 100 | Congruent | .98 | .06 | .95 1.00 |
|  |  |  | Incongruent | .98 | .04 | .95 1.00 |
|  |  | 500 | Congruent | .99 | .02 | .99 1.00 |
|  |  |  | Incongruent | .99 | .02 | .99 1.00 |
|  | PD | 100 | Congruent | .97 | .09 | .95 1.00 |
|  |  |  | Incongruent | .97 | .09 | .94 .99 |
|  |  | 500 | Congruent | 1.00 | .01 | .99 1.01 |
|  |  |  | Incongruent | 1.00 | .02 | .99 1.00 |
| Happy | Control | 100 | Congruent | .97 | .07 | .95 1.00 |
|  |  |  | Incongruent | 1.00 | .09 | .97 1.03 |
|  |  | 500 | Congruent | .99 | .02 | .99 .99 |
|  |  |  | Incongruent | .99 | .02 | .98 1.00 |
|  | PD | 100 | Congruent | .97 | .08 | .94 1.00 |
|  |  |  | Incongruent | .97 | .08 | .94 1.00 |
|  |  | 500 | Congruent | .99 | .02 | .99 1.00 |
|  |  |  | Incongruent | .99 | .02 | .98 1.00 |

| **Effect** | **F(df1,df2 )** | **p-value** | **η_p_^2^** |
| --- | --- | --- | --- |
| Emotion | F(1, 62) = 1.44 | .235 | .023 |
| Duration | F(1, 62) = 4.44 | .039 | .067 |
| Group | F(1, 62) = .19 | .666 | .003 |
| Congruency | F(1, 62) = .30 | .585 | .005 |
| Emotion*Group | F(1, 62) = 1.22 | .274 | .019 |
| Duration*Group | F(1, 62) = .59 | .446 | .009 |
| Congruency*Group | F(1, 62) = 2.99 | .089 | .046 |
| Emotion*Duration | F(1, 62) = 3.37 | .071 | .052 |
| Duration*Congruency | F(1, 62) = 3.89 | .053 | .059 |
| Emotion*Congruency | F(1, 62) = 1.94 | .169 | .030 |
| Duration*Emotion*Congruency | F(1, 62) = 1.86 | .178 | .029 |
| Duration*Congruency*Group | F(1, 62) = 2.03 | .160 | .032 |
| Emotion*Congruency*Group | F(1, 62) = 1.03 | .315 | .016 |
| Duration*Emotion*Group | F(1, 62) = .38 | .539 | .006 |
| Duration*Emotion*Congruency*Group | F(1, 62) = 1.88 | .175 | .029 |

**Main Effects (accuracy)**

| **Group** | **Mean accuracy** | **Standard Error** | **Mean Difference (Standard Error) and p-value** |
| --- | --- | --- | --- |
| Control Group | .99 | .006 | .004 (.009), p = .666 |
| PD | .98 | .006 |  |

| **Duration (ms)** | **Mean accuracy** | **Standard Error** | **Mean Difference (Standard Error) and p-value** |
| --- | --- | --- | --- |
| 100 | .97 | .009 | -.019 (.009), p = .039 |
| 500 | .99 | .001 |  |

| **Congruency** | **Mean accuracy** | **Standard Error** | **Mean Difference (Standard Error) and p-value** |
| --- | --- | --- | --- |
| Congruent | .98 | .005 | -.001 (.002), p = .585 |
| Incongruent | .98 | .005 |  |

| **Emotion** | **Mean accuracy** | **Standard Error** | **Comparison: Mean Difference (Standard Error) and p-value** |
| --- | --- | --- | --- |
| Disgusted | .98 | .005 | vs Fear: -.003 (.002), p = .182  vs Happy: -.004 (.003), p = .704 |
| Fear | .98 | .004 | vs Happy: -.001 (.003), p =1.00 |
| Happy | .99 | .005 |  |

**Group x Duration**

| **Group** | **Duration (ms)** | **Mean**  **accuracy** | **Standard Error** | **95% IC** |
| --- | --- | --- | --- | --- |
| Control | 100 | .98 | .012 | .96 1.00 |
|  | 500 | .99 | .002 | .99 1.00 |
| PD | 100 | .97 | .013 | .94 .99 |
|  | 500 | .99 | .002 | .99 1.00 |

**Group x Emotion**

| **Group** | **Emotion** | **Mean**  **accuracy** | **Standard Error** | **95% IC** |
| --- | --- | --- | --- | --- |
| Control | Disgusted | .98 | .007 | .97 1.00 |
|  | Fear | .99 | .006 | .97 1.00 |
|  | Happy | .99 | .007 | .98 1.00 |
| PD | Disgusted | .98 | .007 | -.97 1.00 |
|  | Fear | .98 | .006 | .97 1.00 |
|  | Happy | .98 | .007 | .97 .99 |

**Group x Congruency**

| **Group** | **Congruency** | **Mean accuracy** | **Standard Error** | **95% IC** |
| --- | --- | --- | --- | --- |
| Control | Congruent | .98 | .007 | .97 1.00 |
|  | Incongruent | .99 | .006 | .98 1.00 |
| PD | Congruent | .97 | .007 | .97 1.00 |
|  | Incongruent | .99 | .007 | .97 .99 |

**Duration x Emotion**

| **Duration (ms)** | **Emotion** | **Mean accuracy** | **Standard Error** | **95% IC** |
| --- | --- | --- | --- | --- |
| 100 | Disgusted | .97 | .010 | .95 .99 |
|  | Fear | .98 | .009 | .96 .99 |
|  | Happy | .98 | .009 | .96 1.00 |
| 500 | Disgusted | .99 | .002 | .99 1.00 |
|  | Fear | .99 | .002 | .99 1.00 |
|  | Happy | .99 | .002 | .99 1.00 |

**Duration x Congruency**

| **Duration (ms)** | **Congruency** | **Mean**  **accuracy** | **Standard Error** | **95% IC** |
| --- | --- | --- | --- | --- |
| 100 | Congruent | .97 | .009 | .95 .99 |
|  | Incongruent | .98 | .009 | .96 1.00 |
| 500 | Congruent | .99 | .001 | .99 1.00 |
|  | Incongruent | .99 | .002 | .97 1.00 |

**Emotion x Congruency**

| **Congruency** | **Emotion** | **Mean accuracy** | **Standard Error** | **95% IC** |
| --- | --- | --- | --- | --- |
| Congruent | Disgusted | .98 | .005 | .97 .99 |
|  | Fear | .99 | .005 | .97 .99 |
|  | Happy | .98 | .005 | .97 .99 |
| Incongruent | Disgusted | .98 | .005 | .97 .99 |
|  | Fear | .98 | .005 | .98 99 |
|  | Happy | .99 | .006 | .98 1.00 |

**Emotion x Group x Duration**

| **Emotion** | **Group** | **Duration (ms)** | **Mean accuracy** | **Standard Error** | **95% CI** |
| --- | --- | --- | --- | --- | --- |
| Disgusted | Control group | 100 | .97 | .014 | .95 1.00 |
|  |  | 500 | .99 | .002 | .99 1.00 |
|  | PD | 100 | .97 | .014 | .94 1.00 |
|  |  | 500 | 1.00 | .002 | .99 1.00 |
| Fear | Control group | 100 | .98 | .013 | .96 1.01 |
|  |  | 500 | .99 | .003 | .99 1.00 |
|  | PD | 100 | .97 | .012 | .95 1.00 |
|  |  | 500 | 1.00 | .002 | .99 1.00 |
| Happy | Control group | 100 | .99 | .013 | .96 1.01 |
|  |  | 500 | .99 | .003 | .99 1.00 |
|  | PD | 100 | .97 | .013 | .94 1.00 |
|  |  | 500 | .99 | .003 | .99 1.00 |

**Emotion x Group x Congruency**

| **Emotion** | **Group** | **Congruency** | **Mean accuracy** | **Standard Error** | **95% CI** |
| --- | --- | --- | --- | --- | --- |
| Disgusted | Control group | Congruent | .98 | .008 | .97 1.00 |
|  |  | Incongruent | .98 | .007 | .97 1.00 |
|  | PD | Congruent | .98 | .008 | .97 1.00 |
|  |  | Incongruent | .98 | .007 | .96 1.00 |
| Fear | Control group | Congruent | .98 | .006 | .97 1.00 |
|  |  | Incongruent | .99 | .006 | .97 1.00 |
|  | PD | Congruent | .99 | .006 | .97 1.00 |
|  |  | Incongruent | .98 | .007 | .97 1.00 |
| Happy | Control group | Congruent | .98 | .007 | .97 1.00 |
|  |  | Incongruent | 1.00 | .008 | .98 1.01 |
|  | PD | Congruent | .98 | .007 | .97 1.00 |
|  |  | Incongruent | .98 | .008 | .96 1.00 |

**Emotion x Duration x Congruency**

| **Emotion** | **Duration (ms)** | **Congruency** | **Mean accuracy** | **Standard Error** | **95% CI** |
| --- | --- | --- | --- | --- | --- |
| Disgusted | 100 | Congruent | .97 | .011 | .95 .99 |
|  |  | Incongruent | .97 | .010 | .95 .99 |
|  | 500 | Congruent | 1.00 | .002 | .99 1.00 |
|  |  | Incongruent | .99 | .002 | .99 1.00 |
| Fear | 100 | Congruent | .98 | .009 | .96 .99 |
|  |  | Incongruent | .97 | .009 | .96 .99 |
|  | 500 | Congruent | .100 | .002 | .99 1.00 |
|  |  | Incongruent | .99 | .003 | .99 1.00 |
| Happy | 100 | Congruent | .97 | .010 | .95 .99 |
|  |  | Incongruent | .99 | .011 | .97 1.00 |
|  | 500 | Congruent | .99 | .002 | .99 1.00 |
|  |  | Incongruent | .99 | .003 | .98 1.00 |

**Group x Duration x Congruency**

| **Group** | **Duration (ms)** | **Congruency** | **Mean accuracy** | **Standard Error** | **95% CI** |
| --- | --- | --- | --- | --- | --- |
| Control | 100 | Congruent | .97 | .013 | .95 1.00 |
|  |  | Incongruent | .99 | .012 | .96 1 .01 |
|  | 500 | Congruent | .99 | .002 | .99 1.00 |
|  |  | Incongruent | .99 | .003 | .99 1.00 |
| PD | 100 | Congruent | .97 | .013 | .94 1.00 |
|  |  | Incongruent | .97 | .013 | .94 .99 |
|  | 500 | Congruent | .100 | .002 | .99 1.00 |
|  |  | Incongruent | .99 | .003 | .99 1.00 |

**Reaction Times**

Normality check: D = .06, p < .001

| **Emotion** | **Group** | **Duration (ms)** | **Congruence** | **Mean RTs** | **Standard Deviation** | **95% CI** |
| --- | --- | --- | --- | --- | --- | --- |
| Disgusted | Control | 100 | Congruent | 548 | 142 | 501 596 |
|  |  |  | Incongruent | 551 | 140 | 503 599 |
|  |  | 500 | Congruent | 559 | 124 | 512 606 |
|  |  |  | Incongruent | 560 | 124 | 515 605 |
|  | PD | 100 | Congruent | 584 | 133 | 534 633 |
|  |  |  | Incongruent | 588 | 137 | 538 638 |
|  |  | 500 | Congruent | 593 | 144 | 545 641 |
|  |  |  | Incongruent | 591 | 136 | 544 637 |
| Fear | Control | 100 | Congruent | 539 | 146 | 489 589 |
|  |  |  | Incongruent | 553 | 144 | 502 604 |
|  |  | 500 | Congruent | 570 | 125 | 525 615 |
|  |  |  | Incongruent | 555 | 126 | 509 600 |
|  | PD | 100 | Congruent | 588 | 142 | 537 640 |
|  |  |  | Incongruent | 593 | 147 | 541 645 |
|  |  | 500 | Congruent | 593 | 133 | 547 640 |
|  |  |  | Incongruent | 590 | 135 | 543 637 |
| Happy | Control | 100 | Congruent | 539 | 140 | 492 586 |
|  |  |  | Incongruent | 552 | 139 | 505 599 |
|  |  | 500 | Congruent | 545 | 121 | 500 590 |
|  |  |  | Incongruent | 557 | 128 | 509 605 |
|  | PD | 100 | Congruent | 582 | 132 | 534 631 |
|  |  |  | Incongruent | 582 | 133 | 533 631 |
|  |  | 500 | Congruent | 590 | 137 | 543 636 |
|  |  |  | Incongruent | 598 | 145 | 548 647 |

| **Effect** | **F(df1,df2 )** | **p-value** | **η_p_^2^** |
| --- | --- | --- | --- |
| Emotion | F(2, 124) = 3.92 | .022 | .059 |
| Duration | F(1, 62) = .20 | .660 | .003 |
| Group | F(1, 62) = 1.77 | .188 | .028 |
| Congruency | F(1, 62) = 4.36 | .041 | .066 |
| Emotion*Group | F(2, 124) = 1.14 | .322 | .018 |
| Duration*Group | F(1, 62) = .01 | .905 | < .001 |
| Congruency*Group | F(1, 62) = .82 | .368 | .013 |
| Emotion*Duration | F(2, 124) = .03 | .968 | .001 |
| Duration*Congruency | F(1, 62) = 3.94 | .052 | .060 |
| Emotion*Congruency | F(2, 124) = 2.99 | .054 | .046 |
| Duration*Emotion*Congruency | F(2, 124) = 5.27 | .006 | .078 |
| Duration*Congruency*Group | F(1, 62) = 1.77 | .189 | .028 |
| Emotion*Congruency*Group | F(2, 124) = 1.10 | .338 | .017 |
| Duration*Emotion*Group | F(2, 124) = 4.22 | .017 | .064 |
| Duration*Emotion*Congruency*Group | F(2, 124) = 1.76 | .176 | .028 |

**Main Effects (reaction times)**

| **Group** | **Mean RTs** | **Standard Error** | **Mean Difference (Standard Error) and p-value** |
| --- | --- | --- | --- |
| Control Group | 552 | 19 | -37 (28), p = .188 |
| PD | 589 | 20 |  |

| **Duration (ms)** | **Mean RTs** | **Standard Error** | **Mean Difference (Standard Error) and p-value** |
| --- | --- | --- | --- |
| 100 | 567 | 17 | -8 (19), p = .660 |
| 500 | 575 | 16 |  |

| **Congruency** | **Mean RTs** | **Standard Error** | **Mean Difference (Standard Error) and p-value** |
| --- | --- | --- | --- |
| Congruent | 569 | 14 | -3.055 (1.46), p = .041 |
| Incongruent | 572 | 14 |  |

| **Emotion** | **Mean RTs** | **Standard Error** | **Comparison: Mean Difference (Standard Error) and p-value** |
| --- | --- | --- | --- |
| Disgusted | 572 | 14 | vs Fear: -.943 (1.76), p = 1.00  vs Happy: 3.610 (1.52), p = .063 |
| Fear | 573 | 14 | vs Happy: 4.553 (1.85), p =.049 |
| Happy | 568 | 14 |  |

**Group x Duration**

| **Group** | **Duration (ms)** | **Mean RTs** | **Standard Error** | **95% IC** |
| --- | --- | --- | --- | --- |
| Control | 100 | 547 | 24 | 499 595 |
|  | 500 | 558 | 23 | 512 603 |
| PD | 100 | 586 | 25 | 537 636 |
|  | 500 | 592 | 24 | 545 639 |

**Group x Emotion**

| **Group** | **Emotion** | **Mean RTs** | **Standard Error** | **95% IC** |
| --- | --- | --- | --- | --- |
| Control | Disgusted | 554 | 19 | 516 593 |
|  | Fear | 554 | 20 | 515 593 |
|  | Happy | 548 | 19 | 510 586 |
| PD | Disgusted | 589 | 20 | 549 629 |
|  | Fear | 591 | 20 | 551 631 |
|  | Happy | 588 | 20 | 548 627 |

**Group x Congruency**

| **Group** | **Congruency** | **Mean RTs** | **Standard Error** | **95% IC** |
| --- | --- | --- | --- | --- |
| Control | Congruent | 550 | 19 | 512 589 |
|  | Incongruent | 555 | 19 | 516 593 |
| PD | Congruent | 588 | 20 | 549 628 |
|  | Incongruent | 590 | 20 | 550 630 |

**Duration x Emotion**

| **Duration (ms)** | **Emotion** | **Mean RTs** | **Standard Error** | **95% IC** |
| --- | --- | --- | --- | --- |
| 100 | Disgusted | 568 | 17 | 533 602 |
|  | Fear | 568 | 18 | 532 604 |
|  | Happy | 564 | 17 | 530 598 |
| 500 | Disgusted | 576 | 16 | 543 608 |
|  | Fear | 577 | 16 | 545 609 |
|  | Happy | 572 | 17 | 539 605 |

**Duration x Congruency**

| **Duration (ms)** | **Congruency** | **Mean RTs** | **Standard Error** | **95% IC** |
| --- | --- | --- | --- | --- |
| 100 | Congruent | 563 | 17 | 529 598 |
|  | Incongruent | 570 | 17 | 535 604 |
| 500 | Congruent | 575 | 16 | 543 607 |
|  | Incongruent | 575 | 17 | 542 608 |

**Emotion x Congruency**

| **Congruency** | **Emotion** | **Mean RTs** | **Standard Error** | **95% IC** |
| --- | --- | --- | --- | --- |
| Congruent | Disgusted | 571 | 14 | 543 599 |
|  | Fear | 573 | 14 | 545 601 |
|  | Happy | 564 | 14 | 537 591 |
| Incongruent | Disgusted | 572 | 14 | 545 600 |
|  | Fear | 573 | 14 | 544 601 |
|  | Happy | 572 | 14 | 544 600 |

**Emotion x Group x Duration**

| **Emotion** | **Group** | **Duration (ms)** | **Mean RTs** | **Standard Error** | **95% CI** |
| --- | --- | --- | --- | --- | --- |
| Disgusted | Control group | 100 | 550 | 24 | 502 597 |
|  |  | 500 | 559 | 23 | 514 605 |
|  | PD | 100 | 586 | 25 | 537 635 |
|  |  | 500 | 592 | 24 | 545 639 |
| Fear | Control group | 100 | 546 | 25 | 496 596 |
|  |  | 500 | 563 | 22 | 518 607 |
|  | PD | 100 | 590 | 26 | 539 642 |
|  |  | 500 | 591 | 23 | 545 638 |
| Happy | Control group | 100 | 545 | 24 | 498 592 |
|  |  | 500 | 551 | 23 | 505 597 |
|  | PD | 100 | 582 | 24 | 534 631 |
|  |  | 500 | 594 | 24 | 546 641 |

**Emotion x Group x Congruency**

| **Emotion** | **Group** | **Congruency** | **Mean RTs** | **Standard Error** | **95% CI** |
| --- | --- | --- | --- | --- | --- |
| Disgusted | Control group | Congruent | 554 | 20 | 515 593 |
|  |  | Incongruent | 555 | 19 | 517 593 |
|  | PD | Congruent | 588 | 20 | 548 629 |
|  |  | Incongruent | 589 | 20 | 550 629 |
| Fear | Control group | Congruent | 555 | 20 | 516 594 |
|  |  | Incongruent | 554 | 20 | 515 593 |
|  | PD | Congruent | 591 | 20 | 550 631 |
|  |  | Incongruent | 591 | 20 | 550 632 |
| Happy | Control group | Congruent | 542 | 19 | 504 580 |
|  |  | Incongruent | 554 | 20 | 515 594 |
|  | PD | Congruent | 586 | 19 | 547 625 |
|  |  | Incongruent | 590 | 20 | 549 630 |

**Emotion x Duration x Congruency**

| **Emotion** | **Duration (ms)** | **Congruency** | **Mean RTs** | **Standard Error** | **95% CI** |
| --- | --- | --- | --- | --- | --- |
| Disgusted | 100 | Congruent | 566 | 17 | 532 600 |
|  |  | Incongruent | 569 | 17 | 535 604 |
|  | 500 | Congruent | 576 | 17 | 543 610 |
|  |  | Incongruent | 575 | 16 | 543 608 |
| Fear | 100 | Congruent | 564 | 18 | 528 600 |
|  |  | Incongruent | 573 | 18 | 537 609 |
|  | 500 | Congruent | 582 | 16 | 549 614 |
|  |  | Incongruent | 572 | 16 | 540 605 |
| Happy | 100 | Congruent | 561 | 17 | 527 595 |
|  |  | Incongruent | 567 | 17 | 533 601 |
|  | 500 | Congruent | 567 | 16 | 535 600 |
|  |  | Incongruent | 577 | 17 | 543 612 |

**Group x Duration x Congruency**

| **Group** | **Duration (ms)** | **Congruency** | **Mean RTs** | **Standard Error** | **95% CI** |
| --- | --- | --- | --- | --- | --- |
| Control | 100 | Congruent | 542 | 24 | 494 590 |
|  |  | Incongruent | 552 | 24 | 504 600 |
|  | 500 | Congruent | 558 | 23 | 513 603 |
|  |  | Incongruent | 557 | 23 | 511 603 |
| PD | 100 | Congruent | 585 | 25 | 535 634 |
|  |  | Incongruent | 588 | 25 | 538 637 |
|  | 500 | Congruent | 592 | 23 | 545 639 |
|  |  | Incongruent | 593 | 24 | 545 640 |

**ABI ANALYSIS**

Normality check: D = .05, p = .25

| **Emotion** | **Group** | **Duration (ms)** | **Mean ABI** | **Standard Deviation** | **95% CI** |
| --- | --- | --- | --- | --- | --- |
| Disgusted | Control group | 100 | 2.47 | 25.93 | -8.00 12.94 |
|  |  | 500 | 2.59 | 25.14 | -6.19 11.36 |
|  | PD | 100 | 4.40 | 33.96 | -6.40 15.20 |
|  |  | 500 | -2.56 | 25.31 | -11.61 6.50 |
| Fear | Control group | 100 | 13.96 | 26.81 | 4.83 23.09 |
|  |  | 500 | -15.50 | 25.52 | -24.74 -6.27 |
|  | PD | 100 | -1.59 | 25.61 | -11.01 7.83 |
|  |  | 500 | -0.68 | 27.59 | -10.21 8.85 |
| Happy | Control group | 100 | 12.52 | 22.60 | 4.03 21.00 |
|  |  | 500 | 12.15 | 29.68 | 1.98 22.32 |
|  | PD | 100 | -0.62 | 26.13 | -9.37 8.13 |
|  |  | 500 | 8.04 | 28.73 | -2.46 18.53 |

| **Effect** | **F(df1,df2 )** | **p-value** | **η_p_^2^** |
| --- | --- | --- | --- |
| Emotion | F(2, 124) = 3.74 | .027 | .057 |
| Duration | F(1, 62) = 2.41 | .126 | .037 |
| Group | F(1, 62) = 1.79 | .186 | .028 |
| Emotion*Group | F(2, 124) = .87 | .420 | .014 |
| Duration*Group | F(1, 62) = 3.42 | .069 | .052 |
| Emotion*Duration | F(2, 124) = 3.78 | .025 | .057 |
| Duration*Emotion*Group | F(2, 118) = 3.89 | .023 | .059 |

**Main Effects**

| **Group** | **Mean ABI** | **Standard Error** | **Mean Difference (Standard Error) and p-value** |
| --- | --- | --- | --- |
| Control Group | 4.70 | 1.84 | 3.553 (2.64), p = .186 |
| PD | 1.17 | 1.89 |  |

| **Duration (ms)** | **Mean ABI** | **Standard Error** | **Mean Difference (Standard Error) and p-value** |
| --- | --- | --- | --- |
| 100 | 5.19 | 1.97 | 4.52 (2.91), p = .126 |
| 500 | 0.67 | 1.96 |  |

| **Emotion** | **Mean ABI** | **Standard Error** | **Comparison: Mean Difference (Standard Error) and p-value** |
| --- | --- | --- | --- |
| Disgusted | 1.72 | 2.40 | vs Fear: 2.68 (3.22), p = 1.00  vs Happy: -6.30 (3.05), p = .130 |
| Fear | -.95 | 2.46 | vs Happy: -8.97 (3.79), p =.064 |
| Happy | 8.02 | 2.18 |  |

**Group x Duration**

| **Group** | **Duration (ms)** | **Mean ABI** | **Standard Error** | **95% IC** |
| --- | --- | --- | --- | --- |
| Control | 100 | 9.65 | 2.74 | 4.18 15.11 |
|  | 500 | -.26 | 2.73 | -5.72 5.21 |
| PD | 100 | .73 | 2.82 | -4.91 6.37 |
|  | 500 | 1.60 | 2.82 | -4.04 7.24 |

**Group x Emotion**

| **Group** | **Emotion** | **Mean ABI** | **Standard Error** | **95% IC** |
| --- | --- | --- | --- | --- |
| Control | Disgusted | 2.53 | 3.34 | -4.15 9.20 |
|  | Fear | -.771 | 3.43 | -7.62 6.08 |
|  | Happy | 12.33 | 3.04 | 6.26 18.40 |
| PD | Disgusted | .92 | 3.45 | -5.97 7.81 |
|  | Fear | -1.14 | 3.54 | -8.21 5.94 |
|  | Happy | 3.71 | 3.13 | -2.55 9.97 |

**Duration x Emotion**

| **Duration (ms)** | **Emotion** | **Mean ABI** | **Standard Error** | **95% IC** |
| --- | --- | --- | --- | --- |
| 100 | Disgusted | 3.44 | 3.76 | -4.09 10.96 |
|  | Fear | 6.18 | 3.28 | -.38 12.74 |
|  | Happy | 5.95 | 3.05 | .15 12.04 |
| 500 | Disgusted | .01 | 3.15 | -6.29 6.32 |
|  | Fear | -8.09 | 3.32 | -14.72 -1.46 |
|  | Happy | 10.09 | 3.66 | 2.79 17.40 |

**AFI ANALYSIS**

Normality check: D = .06, p = .14

| **Emotion** | **Group** | **Duration (ms)** | **Mean AFI** | **Standard Deviation** | **95% CI** |
| --- | --- | --- | --- | --- | --- |
| Disgusted | Control group | 100 | 3.24 | 19.52 | -7.27 13.75 |
|  |  | 500 | -2.69 | 23.21 | -10.80 5.42 |
|  | PD | 100 | -.62 | 38.45 | -11.47 10.22 |
|  |  | 500 | -.89 | 23.42 | -9.26 7.48 |
| Fear | Control group | 100 | 12.53 | 22.51 | 2.67 22.39 |
|  |  | 500 | -15.98 | 27.15 | -25.52 -6.43 |
|  | PD | 100 | -5.08 | 33.46 | -15.26 5.09 |
|  |  | 500 | -.82 | 27.71 | -10.66 9.03 |
| Happy | Control group | 100 | 12.40 | 18.56 | 5.37 19.44 |
|  |  | 500 | 9.39 | 21.63 | 1.29 17.48 |
|  | PD | 100 | .61 | 21.85 | -6.66 7.87 |
|  |  | 500 | 2.72 | 24.89 | -5.64 11.07 |

| **Effect** | **F(df1,df2 )** | **p-value** | **η_p_^2^** |
| --- | --- | --- | --- |
| Emotion | F(2, 124) = 5.69 | .004 | .084 |
| Duration | F(1, 62) = 2.14 | .149 | .033 |
| Group | F(1, 62) = 1.45 | .233 | .023 |
| Emotion*Group | F(2, 124) =1.54 | .218 | .024 |
| Duration*Group | F(1, 62) = 4.13 | .047 | .062 |
| Emotion*Duration | F(2, 124) = 2.79 | .066 | .043 |
| Duration*Emotion*Group | F(2, 118) = 4.64 | .011 | .070 |

**Main Effects**

| **Group** | **Mean AFI** | **Standard Error** | **Mean Difference (Standard Error) and p-value** |
| --- | --- | --- | --- |
| Control Group | 3.15 | 2.21 | 3.83 (3.18), p = .233 |
| PD | -.68 | 2.28 |  |

| **Duration (ms)** | **Mean AFI** | **Standard Error** | **Mean Difference (Standard Error) and p-value** |
| --- | --- | --- | --- |
| 100 | 3.85 | 2.54 | 5.22 (3.57), p = .149 |
| 500 | -1.38 | 2.23 |  |

| **Emotion** | **Mean AFI** | **Standard Error** | **Comparison: Mean Difference (Standard Error) and p-value** |
| --- | --- | --- | --- |
| Disgusted | -.24 | 2.25 | vs Fear: 2.10 (2.79), p = 1.00  vs Happy: -6.52 (2.21), p = .014 |
| Fear | -2.34 | 2.52 | vs Happy: -8.62 (2.94), p =.014 |
| Happy | 6.28 | 1.81 |  |

**Group x Duration**

| **Group** | **Duration (ms)** | **Mean AFI** | **Standard Error** | **95% IC** |
| --- | --- | --- | --- | --- |
| Control | 100 | 9.39 | 3.54 | 2.32 16.46 |
|  | 500 | -3.09 | 3.10 | -9.30 3.11 |
| PD | 100 | -1.70 | 3.65 | -9.00 5.60 |
|  | 500 | .34 | 3.20 | -6.06 6.74 |

**Group X Emotion**

| **Group** | **Emotion** | **Mean AFI** | **Standard Error** | **95% IC** |
| --- | --- | --- | --- | --- |
| Control | Disgusted | .27 | 3.13 | -5.99 6.54 |
|  | Fear | -1.73 | 3.50 | -8.73 5.28 |
|  | Happy | 10.90 | 2.51 | 5.87 15.92 |
| PD | Disgusted | -7.76 | 3.23 | -7.22 5.71 |
|  | Fear | -2.95 | 3.61 | -10.17 4.28 |
|  | Happy | 1.66 | 2.59 | -3.52 6.85 |

**Duration x Emotion**

| **Duration (ms)** | **Emotion** | **Mean AFI** | **Standard Error** | **95% IC** |
| --- | --- | --- | --- | --- |
| 100 | Disgusted | 1.31 | 3.78 | -6.24 8.86 |
|  | Fear | 3.72 | 3.54 | -3.36 10.81 |
|  | Happy | 6.51 | 2.53 | 1.45 11.56 |
| 500 | Disgusted | -1.79 | 2.92 | -7.62 4.04 |
|  | Fear | -8.40 | 3.43 | -15.25 -1.54 |
|  | Happy | 6.05 | 2.91 | .24 11.87 |
